# Facilitating Interprofessional Education in an Online Environment during the COVID-19 Pandemic: A Mixed Method Study

**DOI:** 10.3390/healthcare9050567

**Published:** 2021-05-11

**Authors:** Jitendra Singh, Barbara Matthees

**Affiliations:** School of Nursing & Healthcare Leadership, College of Science, Health, & the Environment, Minnesota State University Moorhead, Moorhead, MN 56563, USA; matthees@mnstate.edu

**Keywords:** interprofessional education (IPE), COVID-19, pandemic, nursing, healthcare, health care, online education, communication, collaboration, mixed methods study

## Abstract

With the COVID-19 crisis and rapid increase in cases, the need for interprofessional education (IPE) and collaborative practice is more important than ever. Instructors and health professionals are exploring innovative methods to deliver IPE programs in online education This paper presents a mixed methods study where an interprofessional education program was delivered/taught using online instruction. Using a survey/questionnaire adapted from the Readiness for Interprofessional Learning Scale (RIPLS) and qualitative discussions, students’ readiness towards online IPE program and the importance of such preparation was examined. Out of two hundred fifteen students who completed the IPE program, one hundred eighty five students from clinical and non-clinical health disciplines responded to the questionnaire (86.04% response rate). Additional qualitative content analysis was conducted on a total of seven hundred and thirty six online discussions. Data analysis across all the four subscales of RIPLS suggests that students felt positively about teamwork and collaboration, and valued opportunities for shared learning with other healthcare students. Qualitative data analysis demonstrated that IPE increases awareness of team members’ roles, enhances communication and collaboration and can lead to better care for COVID-19 patients.

## 1. Introduction

Interprofessional Education (IPE) is defined as “occasions when two or more professions learn with, from and about each other to improve collaboration and the quality of care” [1]. Errors in processes, failure to work as part of a team and lack of a coordinated effort result in medical complications or even patient deaths in healthcare settings. These events not only lead to patient safety issues, but also result in increased costs to the healthcare system [2]. Understanding team members’ roles in patient care processes and a team-based approach that involves collaboration among the different units and departments of an organization can be used to make the system safer for consumers of healthcare services [3]. Interprofessional education and the ability to work in collaboration with other health professionals can lead to reductions in difficulties faced by health organizations in different countries [4]. Teamwork, collaboration, and involvement of employees from different disciplines (who may have been operating in silos) promotes interaction among colleagues, which, in turn, could lead to a fresh look at existing issues/problems and identification of potential solutions to those problems [3,5].

With the global health crisis and rapid increase in COVID cases, there is an increased need for interprofessional education and collaborative programs. As interprofessional practice continues to evolve, instructors and health professionals are exploring innovative methods to deliver IPE programs in online medium of practice. While using online platform/medium for interprofessional learning is a laudable goal for academic institutions [6], there is extremely limited evidence on online IPE programs where clinical and non-clinical health students learn together in a team-based setting. This preparation will help students in undergraduate programs who wish to serve in clinical and/or non-clinical environments in healthcare settings. While clinical disciplines include nursing, athletic training, and other field of study, non-clinical disciplines include (but are not limited to) programs such as health administration, gerontology, social work, and public health. Inclusion of IPE programs during the first year of education will allow students to learn from each other and appreciate what team members can accomplish as a team in a complex healthcare environment.

### Background

As problems posed by the COVID-19 pandemic continue to grow, it is imperative that clinical providers work in close coordination with professionals from different disciplines, departments and sectors of healthcare. Evidence suggests that patients and families experience fragmented care, especially when health professionals are unable to work as one team within healthcare settings. The consequences of failing to work effectively in a team-based setting results in increased costs, inefficiency, lower quality of care and decreased patient satisfaction. Further, failure to work as part of a team and lack of a coordinated effort may result in medical complications or even patient deaths in healthcare settings [7]. Wakely et al. (2013) conducted a study where students from several health disciplines participated in a program that incorporated interprofessional learning modules [8]. It was found that learning about various health disciplines led to improvement in student attitudes (indicated by scores on readiness for interprofessional learning scale). Sani et al. (2011) assessed changes in students’ attitudes after they completed interprofessional learning module. This study reported noteworthy improvements in scores on collaboration and team work [9].

A case study conducted by Evans et al. (2012) examined the effect of inclusion of interprofessional learning modules on students who were studying dentistry and dental technology at the Griffith University. Closer analysis of results suggested that inclusion of interprofessional practices led to positive professional identity and better communication between students [10]. In a quantitative study (Evans et al., 2013), perceptions regarding roles, responsibilities, communication, and team work were compared across two student groups enrolled in dental technology programs. While one group was exposed to a program with content in interprofessional education, other group was a part of traditional program. Results indicated that exposure to interprofessional learning led to changes in attitudes and resulted in improved collaboration among students [11]. Research suggests that interprofessional education and knowledge-sharing among the different disciplines can lead to highly-coordinated, effective patient care in health care settings [12]. This, in turn, can lead to better patient outcomes, increased satisfaction for the provider, and better usage of existing resources. While there are several studies that show importance of interprofessional education programs, it is important to note that health care disciplines still operate in silos and there is a very little opportunity for students in different disciplines to learn together [13].

Research on IPE has focused on clinical programs and traditional on-campus/university based programs. Given that the COVID-19 crisis will continue, academic institutions need to explore new methods to offer IPE and prepare a next generation of leaders who are not afraid to utilize methods that could transform healthcare delivery. There is a scarcity of literature that examines how interprofessional education can be delivered using online medium of instruction [6]. With growth in online education, especially in the field of healthcare, it is important to explore opportunities where professionals from different disciplines can learn together. Using a mixed methods approach, this study aimed to examine students’ readiness and attitudes towards online interprofessional education and importance of such preparation during COVID-19, a public health crisis that has resulted in more than 70 million cases and approximately 1.6 million deaths worldwide.

## 2. Materials and Methods

### 2.1. Participants and Study Design

A total of 215 students, enrolled in an undergraduate healthcare program focused on interprofessional education and learning, were invited to participate in the study. It is important to focus on undergraduate healthcare students because education/training of the majority of these students takes place in silos with a very little chance to collaborate with professionals from different disciplines. An email describing the purpose of the study was sent to all the students. In order to assess impact of IPE training, a pre and post-test methodology was used. All the students were required to complete a survey/questionnaire adapted from Readiness for Interprofessional Learning Scale (RIPLS) prior to beginning of the IPE curriculum and once they finished the IPE curriculum. Qualitative content analysis was completed to analyze students’ responses recorded during IPE program. This provided direct insights into students’ thoughts about collaboration and team-work as they worked in interprofessional teams. The study commenced once approval to conduct the study was obtained from the Institutional Review Board at the university.

### 2.2. Description of IPE Program

The entire IPE educational program was divided into five online modules. These modules were based on the core competencies outlined by Interprofessional Collaborative Practice [14]. The first module focused on introduction of participants and discussion of key concepts in the field of IPE. Student introductions were recorded and used later to form interprofessional teams needed to work on case studies.

In the second module, quality and safety concepts were discussed such as problems related to COVID-19 pandemic, medical complications, and shortage of medical staff. Instructor engaged students in conversations/topics focused on the importance of IPE and need for interprofessional team work required to treat serious COVID patients who suffer from complex medical conditions or conditions that require health care professionals from different disciples to work together.

The third module focused on roles and responsibilities of team members in clinical and administrative processes in healthcare settings. More specifically, content included readings and videos on trainings of providers, importance of engaging family and care givers in the plan of care, and scope of knowledge and abilities of team members as they work on providing safe, high quality and efficient care to patients.

The fourth module focused on the importance of communication between providers during the patient care processes especially in COVID-19 cases. The learning material allowed students to think critically about problems due to faulty communication, fragmented systems of care and adverse events that may lead to patient/resident deaths in facilities. Further, content on utilization of standardized format for communication, importance of respectful communication to patient, providers and families and several communication strategies were included.

The readings and material in the fifth module focused on the importance of team work between providers as they provide care to critical patients in healthcare facilities. Content related to the process of team development, ethical guidelines, shared decision making, problem solving and leadership practices was covered.

### 2.3. Interprofessional Case Studies and Assignments

The entire program included four quizzes, three discussion questions, and two case studies. While quizzes focused on basic understanding of the content, discussion questions and case studies required students to demonstrate in-depth understanding of the material. Case study and discussions focused on scenarios where providers from different disciplines worked as a team when severe cases of COVID-19 were admitted to healthcare facility, roles of providers, team-work, and collaboration. Students completed these cases and discussions via zoom meetings. Students also submitted written response to discussion question via online learning management system. Practitioners from different health disciplines, both clinical and non-clinical, were invited, electronically, to meet with students and present their point of view while dealing with COVID-19 cases.

### 2.4. Quantitative Study Instrument

The Readiness for Interprofessional Learning Scale (RIPLS) was used to collect data for this study. The RIPLS has gained wide acceptance amongst researchers who focus on interprofessional education and learning in healthcare. This scale consists of 19 items, divided into four different subscales. These subscales are as follows: (a) *team work and collaboration* (items 1–9), (b) *negative professional identity* (10–12), (c) *positive professional identity* (13–16), and (d) *roles and responsibilities* [15,16,17]. Researchers have demonstrated statistical validity of the research instrument [18]. Demographic items were added to the scale to collect information about the students.

### 2.5. Quantitative Data Analysis

The data analysis program, IBM SPSS 23.0 was utilized for storing data and analysis. Descriptive statistics were used to examine demographic information. For all the RIPLS subscale, items were summed and scores were calculated. T-tests (independent) were conducted to compare pre-test and post test scores.

## 3. Results

### 3.1. Results of Survey

A total of 185 students responded to the RIPLS (86.04% response rate). Approximately 73% of the participants were females. The median age of the participants was 21 years (IQR = 15) and ranged from 23 to 67 years. The median amount of time spent in their academic program was 10 months and ranged between 1 and 28 months. The participants were mainly white and non-Hispanic or Latino. The majority of the participants worked in healthcare organizations and were from health administration and nursing program (see Table 1).

### 3.2. Comparison of RIPLS Scores

Results of the RIPLS scores demonstrated that there was no significant difference between disciplines/fields of study. More specifically, scores on pre-test (Mean = 69.25 ± 10.06) were not very different from scores on post-test (Mean = 72.12 ± 9.01) across different disciplines’. The RIPLS pre-test score ranged between 67.42 ± 9.32 and 71.54 ± 7.28 with psychology scoring the lowest among the disciplines and athletic training scoring the highest. RIPLS pre-test score for Health Administration was lower than athletic training and health and medical science students. The pre-test score of social work students was lower than health administration students.

The RIPLS post test scores ranged between 68.42 ± 9.34 and 71.94 ± 8.62. There were improvements in groups of students who were from health administration, psychology, and nursing programs. Further, students in gerontology, health and medical sciences and social work demonstrated increase in their post-test scores (see Table 2).

### 3.3. Online Discussions and Qualitative Data Analysis

Online open-ended discussion questions were included as one of the data sources for the study. These open-ended questions allowed students to express their own thoughts. Response time and the amount of text was not restricted and these questions enabled students to explain themselves freely [19] and describe how they felt about teamwork, communication, and shared learning especially during the public health crisis. This can be extremely hard to achieve when questionnaires or surveys are used. Researchers made every effort to reduce bias and did not influence participants’ responses. The students were asked to describe (1) how interprofessional education and team work can be used to enhance patient safety and care for patients suffering with COVID-19, (2) their perceptions regarding how members of interprofessional teams strive to work on clinically complex cases and (3) the importance of awareness of team members’ roles and communication between members while caring for COVID-19 cases.

An inductive approach using content analysis was utilized to conduct the analysis of participants’ discussion posts. A total of seven hundred and thirty six posts were analyzed. Content analysis allows researchers to examine participants’ responses by carefully coding and finding themes in the collected data. It is noteworthy that content analysis has been widely utilized on written texts irrespective of the data collection approach. The principal investigator of the study hired two research assistants to perform data analysis of the study. Both the assistants were trained in research methods and had prior experience in working on qualitative research projects. Upon completion of open coding, researchers independently created codes, subcategories, generic categories, and main categories. Once individual data analysis was completed, the team met to discuss data analysis and reach to final consensus on categories and results [19,20]. The summary of findings are reported here.

### 3.4. Result of Qualitative Content Analysis

The final analysis resulted in generation of 38 codes, 15 sub categories, six generic categories and three main categories. The main categories were (1) IPE increases awareness of team members’ roles and enhances collaboration (2) Increased communication and cohesion among members of teams is critical during the pandemic (3) IPE can lead to better care for COVID-19 patients (See Table 3)

#### 3.4.1. IPE Increases Awareness of Team Members’ Roles and Enhances Collaboration

Interprofessional education and experience as part of a diverse healthcare team allows team members to learn about roles and responsibilities of different practitioners. This allows team members to break past professional barriers and build more collaborative infrastructure.

One of the students noted:

*“Increased communication and training on how to collaborate with other departments/specialties, outside facilities, and better communication between provider and patients would be the most significant methods and approaches to increased patient safety and satisfaction.”*

Using complex COVID cases as example, another student noted, “IPE leads to better communication between different departments in a facility, its affiliated clinics, and between providers and patients. It also makes it easier to forward information to outside facilities or other hospital companies (with appropriate patient authorization). This allows for more of a “whole person” treatment plan vs. segmented treatment plans from each department. Everyone can see what has been done, is currently being done, and what hasn’t been tried yet.”

Furthermore, another participant indicated:

*“One unique feature of the interprofessional education is that the professionals working together are expected to not only be an expert in their own field, but to understand the basics of the disciplines they work with. By understanding the basics of disciplines beyond their own, they can better understand a patient’s condition, and this contributes information to paint a better picture of how to help a patient, leading to better results and better quality.”*

Participants indicated that knowing both one’s role and place amidst the team is an important attribute, as well. By understanding their responsibilities to the team, a professional can fulfill their role effectively and take responsibility for their actions. The shared identity of a team is another key component for success. Being able to trust and work with one another is essential for providing patient care in an efficient manner. 

Another student clearly stated “the balance between the two is perhaps the most important; knowing when one’s role overlaps another, and what matters they should or should not get involved with. Further, making sure the information they are relaying to the team is relevant and will assist other professionals in their jobs.”

Adding to this claim, a student who works as nurse mentioned “in midst of pandemic, I have seen that team members are so rushed and may not demonstrate knowledge of how team works as a cohesive unit. Although each discipline is knowledgeable about their individual role, they are not knowledgeable of other roles and there is a lack of coordination and collaboration. This gap could lead to a delay in the length of stay, errors in communication, and even in safety for the patient.” 

As students completed the IPE program, they clearly indicated that when there is an interprofessional team approach, each discipline works in parallel to the other and crosses over with collaboration and coordination to meet the needs of the patient. This might look like bedside huddles, situational awareness of the whole person and environment, and a well-coordinated series of tasks that feel very safe and flowing to the patient. The patient and caregivers will feel confident in the care of the team and this will increase satisfaction.

#### 3.4.2. Increased Communication and Cohesion among Members of Teams Is Critical during the Pandemic

Students described that IPE leads to an increase in communication and cohesion among team members. This results in improved care for patients and families dealing with COVID-19 crisis.

For example, a student who worked as a nursing home administrator indicated that “communication between healthcare teams is super important when it comes to safety and collaboration. In health care the goal is to care for patients which includes keeping them safe. With interprofessional education/practice we can increase the safety of the patients by all of the nurses, doctors, surgeons, etc. involved with them updating each other on the patient. Some patients have a long journey of medical problems when they are admitted to the facility and having all the care providers working together will ensure them that they know what they are doing.”

Another student reemphasized how a patient suffering with a COVID -19 problem will need to interact with doctors, nurses, long term care workers, pharmacists, physical therapists, and a whole host of other team members throughout the process. The student further added “*if this team of care providers have effective communication and collaboration between them the patient will receive better, more effective quality care. Training health care professionals with different backgrounds and areas of focus to work together and collaborate is where interprofessional learning and education can play a major role. Interprofessional education is when students from different care professions learn from and with each other to improve patient care.*”

Students used various examples to explain their points. One example that is worth noting is the infection control nurse’s role which includes the responsibility for policies/procedures that keep staff, visitors, and patients safe. This student added “*one staff member cannot do it all. These committees arise to complete a task or tasks that one individual could not do on their own. An example of working together is implementation of a policy for patient safety during the current pandemic. Upon arrival to the clinic, masks are passed out to patients who are symptomatic, and they are brought back to be treated quickly to help stop the spread of the virus. Team members have to develop a process through collaboration. They then have to implement it to other staff members through education. Not one person alone can develop a policy that doesn’t affect others around them; interprofessional communication/teamwork is necessary in any healthcare setting*.”

#### 3.4.3. IPE Can Lead to Better Care for COVID-19 Patients

Students recognized the effect of the COVID-19 crisis on healthcare system including personal lives of patients, care providers, and professionals who work in public health and data collection. Students called for collaboration and better training to improve the response to emergency situations such as the current pandemic. To avoid adverse events which could lead to deaths, students felt the need for IPE programs where professionals from multiple disciplines can collaborate and work as a part of team.

For instance, a public health nurse indicated:

*“Interprofessional education can help in dealing with challenges related to the pandemic especially when researchers and data analysts work with providers of clinical services. This would allow us to better plan, prioritize who needs urgent care, coordinate care, identify and deal with gaps and avoid duplication of services. We can use data to identify population with chronic diseases and others who are more vulnerable to the problem”*

Students mentioned how IPE can help in building trust and establishing clear lines of communication between members of the patient care team and families. Supporting this claim, a student in Social Work mentioned “*IPE can help in providing foundation for our response. Important relationships between acute care providers, public health professionals, data collection people, and skilled nursing facilities needs to be built. We should make efforts to understand the needs of the community and then focus our attention on the section of community that needs urgent attention*.”

Students expressed themselves freely and used personal examples to demonstrate that they have seen applications of IPE when their relatives were admitted to the hospital. For example, one of the students indicated that “*interprofessional team not only provided assistance to the patient but they worked collaboratively together to come up with a care plan which best fit our needs. They would meet regularly to compare notes and come up with a schedule of care that would benefit the patient. They were so clear about what they were doing and acted quickly when our patient was admitted.*”

Corroborating previous statements and participants response, another student who works in a frontline position at an acute care hospital mentioned that “*you’d much rather have a team of professionals working together than to just have one doctor try to do all the work and fail. A patient is more likely to feel assured when they see a group of health care professionals collaborating to find a solution. That patient will be satisfied and content with the care they are receiving due to the collaboration.”*

## 4. Discussion

Interprofessional collaboration and communication in healthcare has been shown to improve patient outcomes [21]. It is expected that including experience and practice in interprofessional practice during the professionals’ education leads to more successful interprofessional collaboration in practice. This interprofessional education lays the groundwork for effective practice.

Research has shown that the setting for learning is critical [21]. Ideally, these educational experiences happen in a face to face, live simulation experience with a mixture of professional practice students, guided by expert educators/practitioners. Research has shown that students’ learning improves as carefully developed interdisciplinary simulations mimic reality closely [22]. However, in the current state of COVID-19 restrictions, live experiences in simulation or a clinical setting may not be easy to perform. At the same time, interprofessional collaboration has never been more important. An alternative educational format becomes necessary to accomplish the outcome of practice professionals entering the clinical arena with interprofessional communication and collaboration skills.

Online education provides access to programs for many working professionals, especially those distant from the educational setting or unable to attend ‘class’ due to work schedules or other constraints. Indeed, much of higher and professional education has moved to online settings, even prior to the advent of COVID. With the arrival of the pandemic, a great majority of coursework and clinical experiences pivoted rapidly into an online delivery methodology. Usage of e-learning tools has not been consistent across different health profession programs and countries [23]. Recent studies suggested that inclusion of mobile applications and virtual hospitals were well received by students [23,24,25]. It has also been suggested that if used correctly, e-learning can enhance teaching and learning methods in clinical programs [23]. The questions then become whether IPE can be done in an online setting, and whether it is effective in practitioner development.

This study provides important insight into the opportunities that online asynchronous education offers for healthcare students’ understanding of their interprofessional roles. Both quantitative and qualitative methodologies were utilized. Use of a mixed-methods approach may address the ‘what’ and ‘how’ of an IPE intervention and its outcomes [21].

This online IPE program focused on four areas: key concepts in the field of IPE, quality and safety, roles and responsibilities of the various providers, and the importance of communication and teamwork. Several different health-related majors were represented by the enrolled students, which is consistent with professional practice. A variety of educational tools including quizzes, discussion questions, and case studies, are reflected in this mixed methods study.

The quantitative Readiness for Interprofessional Learning Scale (RIPLS) found that students clearly reflected the positive outcomes of IPE, even in the online setting. Qualitatively, students responded to discussion questions and described that:

(1) IPE increases awareness of team members’ roles and enhances collaboration
(2) Increased communication and cohesion among members of teams is critical during the pandemic and
(3) IPE can lead to better care for COVID-19 patients.

These RIPLS quantitative results and qualitative student perspectives provide optimism that IPE can be learned effectively in an online environment, which will be critical moving forward as healthcare aims to improve care through interprofessional collaboration, even in an online, asynchronous environment. This study is unique in that the students were entirely online during a pandemic and represented a variety of healthcare roles. More work needs to be done to expand the understanding of the most effective online teaching/learning tools. Follow up with program graduates will be significant as they move into their professional positions.

## 5. Limitations

Because many students were working in healthcare and other institutions, they did not check their university email regularly. As a result, faculty members struggled to reach out to students and communicate in a timely fashion. This program was offered at a single academic institution located in the Midwest US. While findings and methodology can be utilized at other institutions, these findings may not be generalizable across the US or the globe. COVID-19 pandemic academic management posed additional problems for the implementation of the program. It was hard to invite healthcare practitioners to the class as they were extremely busy in dealing with COVID cases in their roles. As online education in healthcare disciplines continue to grow, more research is needed that can explore students attitudes towards online IPE program across several university campuses.

## 6. Conclusions

The aim of this mixed method study was to examine attitudes and readiness towards interprofessional education and significance of this preparation during the pandemic. Findings suggest that students from both clinical and non-clinical programs valued opportunities for learning together and felt that IPE enhances awareness of team members’ roles and responsibilities and improves collaboration. Participants further suggested that IPE increases communication and cohesion, and results in better care of patients’ which is extremely important while working with COVID patients. Further studies across medical centers and academic settings are needed to explore attitudes of students towards IPE programs. Inclusion of different education settings and additional clinical and non-clinical programs not currently represented in the study sample will allow better understanding of IPE especially while working with patients during public health crisis.

## Figures and Tables

**Table 1:** Characteristic of Sample (*N* = 185).

Subscale Scores	*Mdn.*	*IQR*	*f*(*n*)	%
Age (Years)	21	15		
Time in education program (Months)	10	12		
Sex				
Male			50	27
Female			135	73
Race				
White/Caucasian			142	76
Black/African American			26	14
Asian			6	3
Native Hawaiian or Pacific Islander			0	0
American Indian or Native America			3	1.6
Other			8	4.3
Ethnicity				
Hispanic or Latino			16	8.64
Non-Hispanic or Latino			169	91.35
Education Program				
Health Services Administration (HSAD)			108	58
Nursing			20	10.8
Social Work			17	9.1
Health & Medical Sciences			12	6.48
Psychology			14	7.56
Athletic Training			5	2.7
Gerontology			9	4.8
Work place				
Health care			125	67.56
Education			18	9.7
Other			42	22.7

**Table 2:** Comparison of Scores.

Education Program	Pre-Test Scores (Mean ± SD)	Post-Test Scores (Mean ± SD)	*p* Value
Health Administration	69.25 ± 8.42	70.24 ± 9.64	0.73
Nursing	67.42 ± 9.32	70.68 ± 9.21	0.68
Social Work Health and Medical Sciences	68.21 ± 8.63 70.21 ± 8.34	68.42 ± 9.34 70.12 ± 8.68	0.83 0.50
Psychology	66.54 ± 9.45	69.32 ± 9.67	0.64
Athletic Training Gerontology	71.54 ±7.28 69.41 ± 9.48	71.94 ± 8.62 70.42 ± 8.54	0.76 0.89

**Table 3:** Categories.

Category 1	Category 2	Category 3
Awareness of roles and collaboration	Communication and cohesion	Better care for patients

## Data Availability

Data available on request due to restrictions.
